# Biobased Waterborne Polyurethane-Ureas Modified with POSS-OH for Fluorine-Free Hydrophobic Textile Coatings

**DOI:** 10.3390/polym13203526

**Published:** 2021-10-13

**Authors:** Amado Lacruz, Mireia Salvador, Miren Blanco, Karmele Vidal, Amaia M. Goitandia, Lenka Martinková, Martin Kyselka, Antxon Martínez de Ilarduya

**Affiliations:** 1 Color Center, S.A. Ptge. Marie Curie 3, Nau 6, 08223 Terrassa, Spain; msalvador@colorcenter.es; 2 Departament d’Enginyeria Química, Universitat Politècnica de Catalunya, ETSEIB, Diagonal 647, 08028 Barcelona, Spain; antxon.martinez.de.ilarduia@upc.edu; 3 Tekniker, Basque Research and Technology Alliance (BRTA), Surface Chemistry and Nanotechnology Unit, Iñaki Goenaga 5, 20600 Eibar, Spain; miren.blanco@tekniker.es (M.B.); karmele.vidal@tekniker.es (K.V.); amaia.martinez@tekniker.es (A.M.G.); 4 Inotex Spol. s r.o, Stefanikova 1208, 54401 Dvůr Králové nad Labem, Czech Republic; martinkova@inotex.cz (L.M.); kyselka@inotex.cz (M.K.)

**Keywords:** waterproof, water-column, fluorine-free, bio-based, hydrophobic, hybrid POSS, technical textiles, multifunctional fabrics, textile coatings

## Abstract

Waterborne polyurethane-urea dispersions (WPUD), which are based on fully biobased amorphous polyester polyol and isophorone diisocyanate (IPDI), have been successfully synthesized obtaining a finishing agent that provides textiles with an enhanced hydrophobicity and water column. Grafting of trans-cyclohexanediol isobutyl POSS (POSS-OH) to the biobased polymer backbone has also been investigated for the first time and its properties compared to a standard chain extender, 1,3-propanediol (PDO). The chemical structure of WPUD has been characterized by Fourier-transform infrared spectroscopy (FTIR) and nuclear magnetic resonance (NMR). The thermal properties have been evaluated by differential scanning calorimetry (DSC) and thermogravimetric analysis (TGA). Mechanical properties have been studied by tensile stress–strain analysis. Moreover, the particle size, particle size distribution (PSD), and stability of developed waterborne dispersions have been assessed by dynamic light scattering (DLS), Z-potential, storage aging tests, and accelerated aging tests by analytical centrifuge (LUM). Subsequently, selected fabrics have been face-coated by the WPUD using the knife coating method and their properties have been assessed by measuring the water contact angle (WCA), oil contact angle (OCA), water column, fabric stiffness, air permeability, and water vapor resistance (breathability). Finally, the surface morphology and elemental composition of uncoated and coated fabrics have been studied by scanning electron microscopy (SEM) and energy-dispersive X-ray spectroscopy (EDS), respectively. All of the synthesized polyurethane-ureas provided the coated substrates with a remarkable hydrophobicity and water column, resulting in a more sustainable alternative to waterproof coatings based on fluoropolymers, such as PTFE. Grafting POSS-OH to the polymeric backbone has led to textile coatings with enhanced hydrophobicity, maintaining thermal, mechanical, and water column properties, giving rise to multifunctional coatings that are highly demanded in protective workwear and technical textiles.

## 1. Introduction

Our current society relies predominantly on fossil resources such as oil, natural gas, or coal to meet all the needs of modern life. In addition, advanced textile properties such as repellency to liquids or a water column currently require chemicals that are considered of high concern, like fluorochemicals that dominate the water- and oil-repellent textile finishing market because of their excellent performance. However, fluorinated chemicals release harmful substances, giving rise to serious damage to ecosystems worldwide [1,2]. This is not a sustainable situation, and therefore a transition to a less harmful and biobased solution is necessary [3].

Accordingly, sustainability is nowadays an important topic not only for the chemical industry but also for other industrial sectors as well as governments and society itself. Renewable resources derived from plants can be used to produce building blocks suitable for obtaining partially or fully biobased high-performance polymeric materials. Nevertheless, much work remains to be done in this field through innovation and joint efforts involving industries, universities, research centers, and governments. Biobased polymers are a group of materials that are totally or partially derived from biomass. They try to fulfill global trends toward environmentally friendly solutions in the field of electronics, packaging, automobiles, textiles, etc. At present, thanks to the growing supply of commercially available biobased building blocks [4], a great opportunity opens up for the synthesis of sustainable high-performance polymers offering highly demanded features like outstanding mechanical properties, barrier properties, hydrophobicity, and stability against oxidation, hydrolysis, heat, or UV radiation. Biobased polyurethanes are an important group of materials that is attracting growing interest from industry and research groups [5,6]. Chemical, mechanical, and barrier properties of these types of materials can be precisely modulated from the appropriate selection of the starting building blocks and the synthesis strategy [7,8].

In this work, the commercial fully biobased amorphous polyester polyol Priplast 3238 has been combined with IPDI, internal emulsifier DMBA, and chain extenders 1,3-propanediol (PDO), trans-cyclohexanediol isobutyl POSS (POSS-OH), and ethylenediamine (EDA) to obtain a series of waterborne polyurethane-urea dispersions (WPUD). These WPUD have been specifically designed as sustainable fluorine-free coating agents for technical textiles where hydrophobic and liquid barrier finishes are required. A ternary mixture experimental design methodology has been used as a systematic approach to explore the relationship between the polymer structure and the properties of the obtained polymer films and coated fabrics.

**Figure 1:**
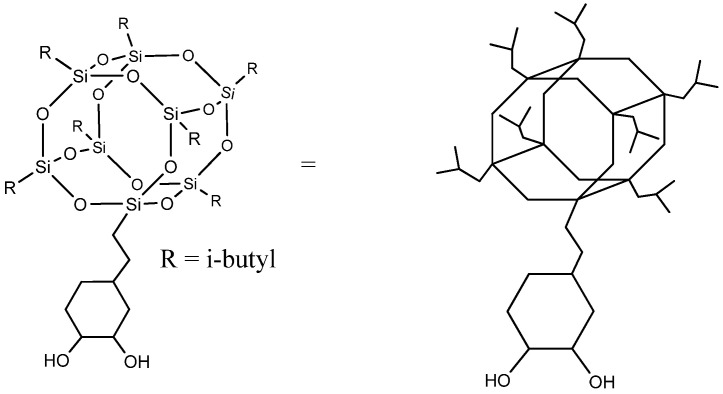
Cyclohexanediol isobutyl POSS (POSS-OH).

Despite reactive polyhedral oligomeric silsesquioxanes having been previously reported in the literature as a building block for polyurethanes [9,10], we describe for the first time the grafting of POSS-OH to a biobased polyurethane-urea. POSS-OH is a hybrid molecule with an inorganic silsesquioxane at the core, organic isobutyl groups attached at seven corners of the cage, and a trans-cyclohexanediol group on the eighth corner that allows its incorporation as a co-monomer in the polyurethane-urea chain. Its structure is displayed in Figure 1. The bulky and highly hydrophobic heptaisobutyl silsesquioxane group is able to confer enhanced hydrophobicity without noticeably affecting the mechanical and thermal properties of the coatings.

## 2. Materials and Methods

### 2.1. Materials

**Figure 2:**
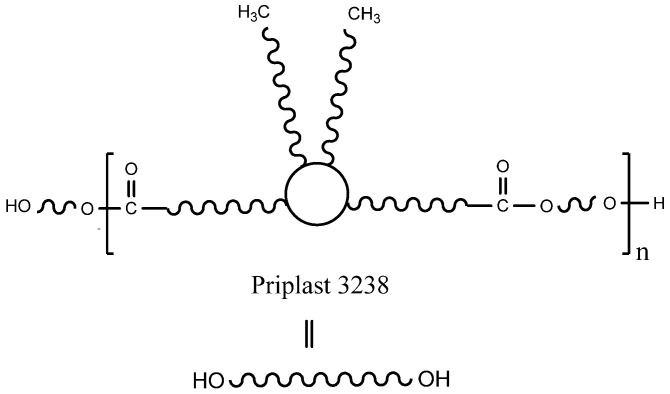
The chemical structure of Priplast 3238.

Fully biobased amorphous polyester polyol based on dimerized fatty acids (Figure 2), commercially known as Priplast 3238 and supplied by Croda Iberica, S.A. (Barcelona, Spain), was used as a main biobased polyol for the synthesis of all of the polyurethane-ureas. Priplast 3238 is a highly hydrophobic, amorphous polyester polyol providing high flexibility at low temperatures, good hydrolytic stability, and enhanced adhesion to dissimilar substrates. This polyol has a *M*_n_ of 2000 g mol^−1^, f(OH) = 2 and an average hydroxyl value of 56 mg KOH g^−1^. Isophorone diisocyanate (IPDI, ≥99.5% purity) was supplied by Evonik Industries GmbH (Essen, Germany). 2,2-Bis(hydroxymethyl)butyric acid (DMBA, 99% purity) was supplied by Anhui Sinograce Chemical CO., LTD (Hefei, People’s Republic of China). 1,3-Propanediol (PDO, ≥99% purity and fully biobased) was supplied by DuPont Tate & Lyle BioProducts (Loudon, TN, USA). Trans-cyclohexanediol isobutyl POSS (POSS-OH) was supplied by Hybrid Plastic Co. Inc. (Hattiesburg, MS, USA) Acetone (≥99.5% purity), triethylamine (TEA, 99% purity), and ethylenediamine (EDA, ≥99% purity) were purchased from Sigma-Aldrich (Darmstadt, Germany). Everchem Specialty Chemicals (Media, PA, USA) supplied Bicat 8108 (bismuth neodecanoate 20%).

The additives to formulate the printing pastes were supplied by Color Center, S.A. (Terrassa, Spain) and are: Defoamer PR (defoamer, mineral oil-based), Complex DG (diethylene glycol-based, runnability improver), DMEA (dimethylethanolamine, neutralizing agent), and Thickener L-120 (polyacrylic acid, associative thickener).

Pristine polyester fabric (UPRON) was supplied by HEDVA a.s. (Moravská Třebová, Czech Republic) and was used as a substrate to perform the coatings. The main characteristics of the pristine UPRON fabric are summarized as follows: 100% polyester fabric (PET, polyethylene terephthalate), plain weave, 172 ± 5 g m^−2^; threads per cm: warp 32 ± 2, weft 14 ± 1.

### 2.2. Synthesis of Waterborne Polyurethane-Urea Dispersions (WPUD)

Priplast 3238 was dried in a vacuum at 110 °C and 35 mbar for 1 h before use. It was introduced into a 750 mL five-necked reactor equipped with a mechanical stirrer, an inlet for nitrogen, and a reflux condenser. DMBA internal emulsifier and POSS-OH or PDO were added to the reactor and degassed for 1 h at 80 °C under stirring to complete homogenization. IPDI and Bicat 8108 catalyst (25 mg per kg of mixture) were subsequently added to the reactor. The temperature was maintained at 80 °C under nitrogen blanketing until the theoretical value of NCO was reached (measured by titration using the dibutylamine method). The reaction time for the prepolymer formation was between 2 and 2.5 h. All of the prepolymers were prepared at an isocyanate/hydroxyl ratio (NCO/OH) of 1.62. Afterward, acetone was added to decrease the viscosity of the prepolymer and facilitate its subsequent dispersion in water (typically 100 g/200 g of prepolymer). The reaction mixture was cooled to 40 °C and TEA was added slowly through a dropping funnel and the mixture maintained for 30 min under stirring to ensure complete neutralization of the carboxyl groups from DMBA. The reaction system was subsequently cooled to 30 °C and cold deionized water at 8 °C was quickly added with vigorous stirring to promote phase inversion, thus obtaining a milky dispersion of the prepolymer in water. Chain extension agent EDA was stoichiometrically added to react with the free isocyanate groups of the prepolymer, previously diluted in water to 20%, drop-by-drop, by keeping the temperature of the mixture between 15 and 18 °C along with gentle stirring for an additional 1 h. Finally, the biobased WPUD was obtained after removing acetone by a rotary evaporator under reduced pressure (250 mbar) at 60 °C. All of the synthesized WPUD were adjusted to a solid content of 35%. It is worth noting that acetone can be readily recovered and recycled.

**Figure 3:**
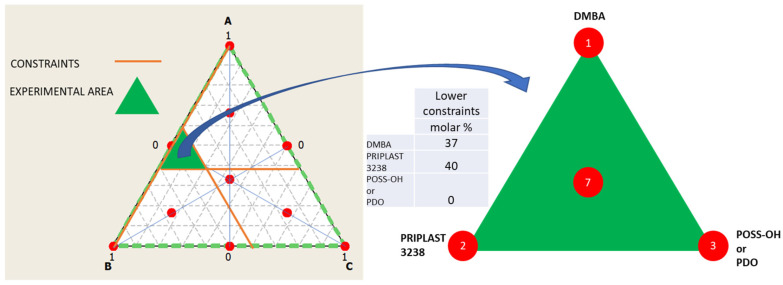
Ternary mixture design of experiments employed to study the proportion between the tree diols that form part of the polymer. **Left**: full design surface area in white with all the theoretical experimental points marked in red and studied area in green, which is delimited by the lower constraints stablished for component A (DMBA) and B (Priplast 3238). Middle: table summarizing the lower constraints stablished for the diols. **Right**: area studied and experimental points of the simplex design plot (1, 2, 3, 7).

The ratio between the three diols used in the prepolymer synthesis (Priplast 3238, DMBA, and POSS-OH or PDO) was systematically varied using the methodology of ternary mixture design of experiments (Figure 3). In our particular case, two experimental designs were established, in the first design the ternary mixture of DMBA, Priplast 3238 and POSS-OH was investigated and in the second design the ternary mixture of DMBA, Priplast 3238 and PDO was investigated. Experiments 1 and 2 were common to both experimental designs as they did not contain PDO or POSS-OH. Lower constraints were applied to DMBA and Priplast 3238 because, due to the particularity of the system, it is not possible to study the proportions of the three diols in the full range (from 0 to 100 molar%). For example, the amount of DMBA could not be equal to 0, since then the final polymer would not be dispersible in water; therefore, the lower constraint for DMBA was established in 37 molar%. A minimum amount of Priplast 3238 was also established so that all of the prepolymers contained at least a 40 molar% of this biobased polyol.

Table 1 shows the simplex design table with all of the performed experimental points from the simplex ternary mixture design. The six columns on the right show the molar% of each polyol employed in the synthesis considering the lower constraints, hard segment content in wt.%, and bio-based content of each experiment in wt.%, respectively.

Polyurethane-urea films suitable for NMR, mechanical, thermal, and swelling characterization were prepared by carefully pouring 20 g of WPUD in a circular Teflon mold, allowing the water to evaporate slowly at 40 °C into a vented oven for 48 h to obtain 1-mm thick films that were subsequently cured and dried at 60 °C for 48 h in a vacuum oven.

### 2.3. Application of WPUD on Fabrics by Coating

The fabrics were coated by printing pastes that were made of WPUD, water, defoamer, runnability improver, DMEA, and thickener (see Table 2 for the standard printing paste formulation, solid content, and viscosity).

The knife coating procedure, which is a widely used coating method in the textile sector, was employed to coat all of the fabrics with the corresponding printing pastes. Knife coating was performed using a laboratory coating machine R2R continuous-line Werner Mathis in a coating regime, air knife at 90 °, followed by drying in a vented oven at 110 °C at a speed of 1 m per min^−1^ and curing at 150 °C at a speed of 0.4 m per min^−1^. The coating procedure on glass slides has been done by a more convenient manual Quadrangular Applicator with a gap of 60 µm, followed by drying at 90 °C for 5 min and curing at 120 °C for 2 min.

The dry add-on of the coated fabrics was calculated as follows: a sample cutter James H. Heal model 230/100 was used to cut out regular circular specimens of a fixed area (100 cm^2^) from the uncoated fabric and all of the coated fabrics. The calculation of weight per square meter (grammage, G) of a given specimen was performed by multiplying the specimen’s weight measured by a balance with a readability of 0.01 g by a factor of 100. Finally, the dry add-on of a coated fabric was calculated using Equation (1), where Gc and Gu are the grammages of the coated and uncoated specimens, respectively.

Dry add on = Gc − Gu (1)

### 2.4. Characterization Techniques

#### 2.4.1. Characterization of Synthesized Polymers and Dispersions

Fourier-transform infrared spectroscopy (FTIR) was employed for chemical characterization of the WPUD. The FTIR spectra were obtained using a Perkin Elmer Spectrum Two spectrometer (Madrid, Spain) equipped with transmission accessory. A drop of each waterborne dispersion was spread on a SeZn FTIR window and then dried under an IR lamp to evaporate water and to obtain a thin film. Eight scans were taken for each sample in the range of 4000–500 cm^−1^ with a resolution of 4 cm^−1^.

The structural characterization of all the WPUD casted films was performed by ^1^H-NMR using a Bruker AMX-300 spectrometer (Billerica, MA, USA) at 25 °C operating at 300.1 MHz. Samples were dissolved in deuterated chloroform or deuterated tetrachloroethane, and spectra were internally referenced to tetramethylsilane (TMS). Approximately 10 mg of sample dissolved in 1 mL of solvent was used to collect the ^1^H-NMR spectra. Sixty-four scans were acquired with 32 K data points as well as a relaxation delay of 1 s.

Differential scanning calorimetry (DSC) studies of the dry films of all the synthesized WPUD as well as Priplast 3228 were carried out to determine first- and second-order thermal transitions. *T*_g_ and *T*_m_ values were determined by heating the sample from −90 to 200 °C at a constant heating rate of 10 °C min^−1^ in a Mettler-Toledo DSC1 module (Gieβen, Germany) equipped with an intracooler and previously calibrated with high purity indium and zinc standards. Experiments were conducted in a dry atmosphere, under a nitrogen constant flow of 50 mL/min, working with around 10 mg samples and using microperforated aluminum pans.

The thermal stability of the synthesized WPUD films was studied by thermogravimetric analysis (TGA) using a Mettler-Toledo TGA2 module (Columbus, OH, USA). The thermogravimetric analysis consisted of recording the weight loss of the samples that were subjected to a temperature gradient from 25 °C to 600 °C at 10 °C min^−1^. TGA curves were recorded under nitrogen and air atmospheres.

Mechanical properties were determined by stress–strain tensile measurements. The tests were carried out following the BS ISO 37: 2005 standard using a Zwick/Roell model 500 N (Ulm, Germany). The measurements were carried out on dumbbell-shaped Type 4 specimens cut from the WPUD films. The test conditions were as follows: preload 0.1 MPa, preload speed 1 mm min^−1^, and test speed 50 mm min^−1^. For each WPUD, at least five samples were taken in different parts of the films and tested. The characteristic parameters measured were: elastic modulus (E), stress at break (σ_b_), deformation at break (ε_b_), and stress at 100% strain (σ_100%_).

Water-swelling measurements were performed using dumbbell-shaped Type 4 specimens cut from the WPUD films. The samples were placed in a closed vial with 20 mL of deionized water at 25 °C for 48 h. Swelling degree was determined by Equation (2), where w_0_ and w were respectively the weight of the initial dried material and of the swollen material after 48 h. The experiments were carried out in triplicate for each specimen.

(2) $$\mathrm{Swelling}\;\left(\%\right)=\frac{w-w_{0}}{w_{0}}\times 100$$

Moreover, the particle size and particle size distribution (PSD) of developed aqueous polymeric dispersions were characterized via dynamic light scattering (DLS). The stability of the dispersions was analyzed using Z-potential measurements. DLS and Z-potential tests were performed on a Malvern Zetasizer ZS at 20 °C. Particle size and Z-potential measurements were performed after the dilution of the WPUD to 1 wt.% with deionized water buffered at pH 8.2. Storage stability was assessed for all of the WPUD stored in sealed glass vials and kept for 6 months at 4 °C and 40 °C, respectively. Finally, the WPUD specimens were subjected to accelerated sedimentation tests using an analytical centrifuge, LUMiFuge 110–153.3–12 (LUM GmbH, Berlin, Germany), in order to evaluate long-term stability. In each measurement, the suspension was pipetted into a polyamide transparent cell with a path length of 2 mm. Thereafter, a transmission profile at 470 nm of the samples was analyzed at 4 °C and 40 °C and a relative centrifugal force of 2000× *g* with a scanning rate of once every 60 s for 5 h.

#### 2.4.2. Characterization of Coated Textiles

The water and oil repellence of the coated fabrics was tested by measuring the contact angle of a droplet of water and a droplet of olive oil placed on the surface of the coated textile, respectively. Water contact angle (WCA) and oil contact angle (OCA) measurements were carried out under ambient conditions with a SURFTENS Universal automatic goniometer (OEG GmbH, Frankfurt, Germany). Static contact angle of the air–liquid interface was measured on each coated fabric. The volume of each liquid droplet was 5 μL and the average value of five measurements, made at different positions of the textile surface, was adopted as the value of WCA or OCA.

Hydrostatic pressure, fabric stiffness, air permeability, and water vapor resistance were evaluated according to the indicated standards in Table 3.

In ISO 11092, the water vapor resistance of the fabric (R_et_) refers to the ratio of the water vapor pressure difference on both sides of the fabric (P1, P2) to the heat flux evaporated vertically (Q) per unit area of the fabric (S). The units of R_et_ are square meters pascal per watt, m²·Pa W^−1^.

(3) $$\mathrm{Ret}=\frac{S\;\left(P1-P2\right)}{Q}$$

Finally, the surface morphologies of the relevant coated and uncoated fabrics were studied via scanning electron microscopy (SEM) using an Ultra Gemini-II microscope from Carl Zeiss SMT (LLC, Thornwood, NY, USA), also equipped with energy-dispersive X-ray spectroscopy (EDS), which was employed for element analysis on textile surfaces.

## 3. Results and Discussion

### 3.1. Synthesis and Characterization of WPUD

**Figure 4:**
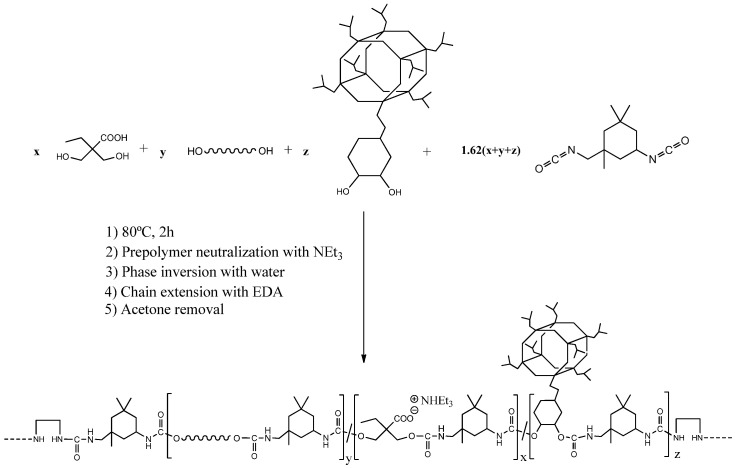
Synthesis scheme of the waterborne polyurethane-urea dispersions using POSS-OH as a co-polyol.

A series of WPUD were synthesized using the prepolymer method, as reported in the experimental section. Once the prepolymer was obtained, the neutralization of the carboxyl groups with triethylamine and subsequent phase inversion in water was carried out. Finally, chain extension via EDA was performed, followed by acetone removal by rotary evaporation and standardization to 35% solid content. The scheme of the synthesis strategy of the WPUD can be seen in Figure 4.

**Figure 5:**
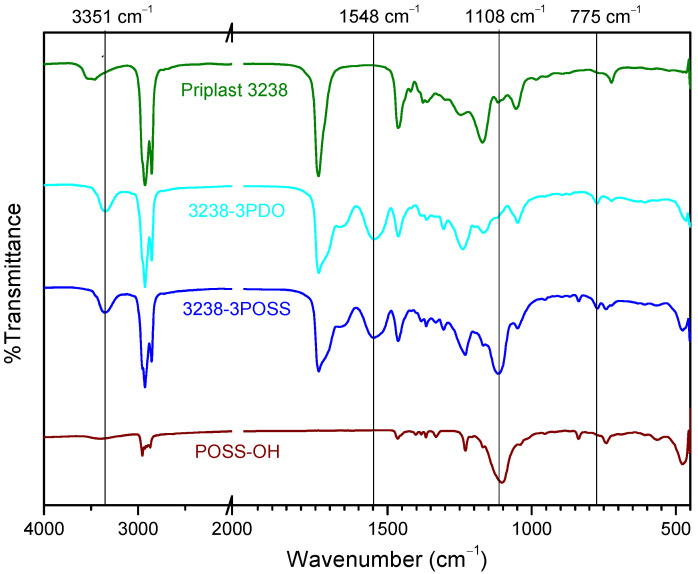
FTIR spectra of (from top to bottom): Priplast 3238, 3238-3PDO, 3238-3POSS, and pure POSS-OH.

WPUD were first characterized by FTIR. The infrared spectra of all synthesized polymers showed the complete conversion of isocyanate groups judging by the absence of the characteristic free isocyanate band at 2275 cm^−1^. By comparing the spectrum of the starting polyol Priplast 3238 with those of the synthesized polyurethane-ureas, the appearance of new bands that are indicative of the formation of urethane/urea bonds could be clearly confirmed (Figure 5). The broad band of NH asymmetrical and symmetrical stretching vibration at 3351 cm^−1^ indicated the great extent of NH established hydrogen bonds with carbonyl groups from urethane, urea, ester, and ionic carboxylate from internal emulsifier DMBA [11]. The region of 1500–1600 cm^−1^ showed NH-CO stretching and NH bending bands of the urethane group at 1548 cm^−1^. The region between 1740 and 1600 cm^−1^ showed characteristic C=O stretching bands from DMBA carboxylate, urethane, and urea carbonyl groups at approximately the 1661–1700 cm^−1^ region, partially overlapped with the ester band from the polyester polyol at 1739 cm^−1^. COC(O) stretching bands and NH out-of-plane bending bands from urethane functional groups at 1242 cm^−1^ and 775 cm^−1^, respectively, were clearly observed. On the other hand, the pure POSS-OH showed strong bands at 1108, 743, and 480 cm^−1^ that corresponded to the Si-O-Si asymmetric stretching, symmetric stretching, and bending mode, respectively. The pure POSS-OH also showed a band at 1039 cm^−1^ that, according to [9], can be attributed to Si-isobutyl. 3238-3PDO, 3238-1, 3238-2, 32387PDO showed all the aforementioned characteristic polyurethane-urea bands while 3238-3POSS and 3238-7POSS showed the same bands plus the characteristic silsesquioxane peaks.

**Figure 6:**
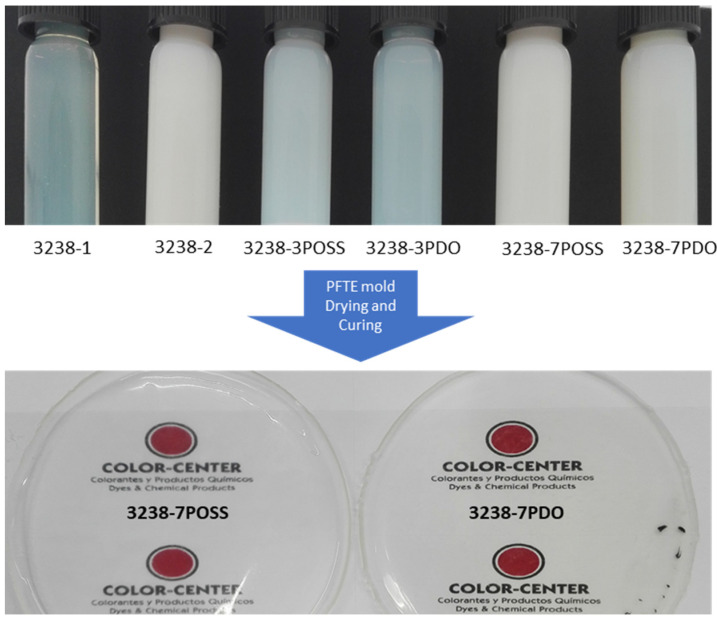
Appearance of the synthesized WPUD (**top**) and casted films (**bottom**) from two of the polyurethane-urea dispersions (3238-7POSS and 3238-7PDO).

All of the synthesized WPUD were dried on Teflon plates to obtain the corresponding films. All of the obtained films were transparent (except the ones containing POSS that were not completely clear) and homogenous and were used for NMR, mechanical, thermal, and swelling characterization. Figure 6 shows the appearance of the synthesized WPUD as well as the appearance of casted film from two of the polyurethane-urea dispersions (3238-7POSS and 3238-7PDO).

**Figure 7:**
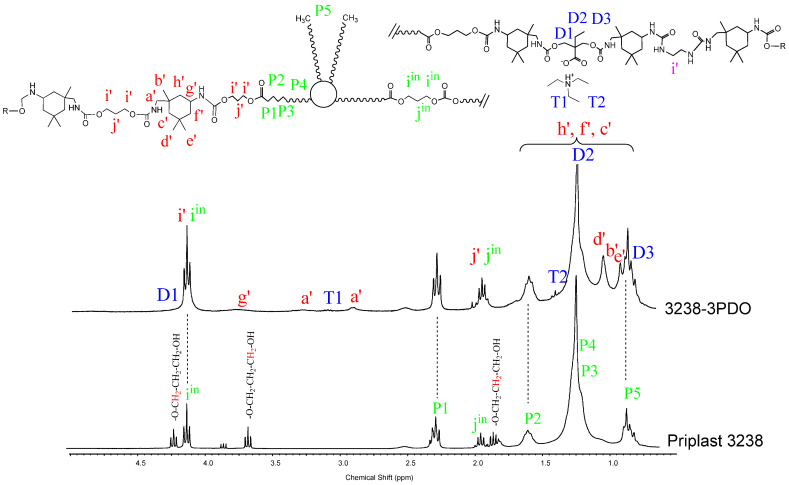
^1^H NMR of experiment 3238-3PDO and starting polyol Priplast 3238 with peak assignments.

**Figure 8:**
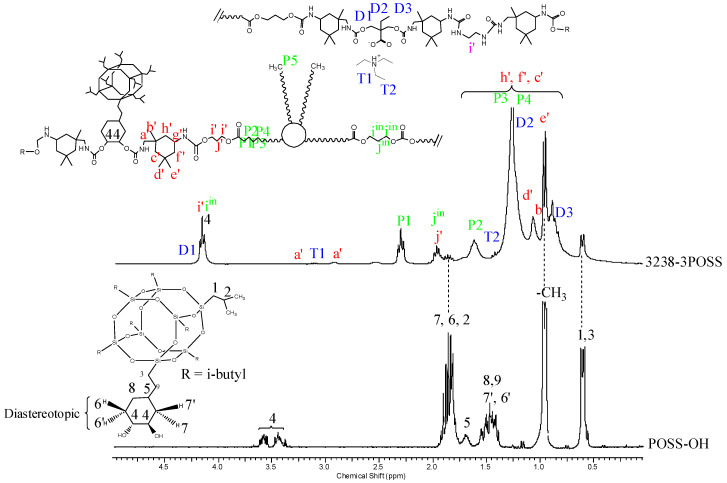
^1^H NMR of experiment 3238-3POSS and pure POSS-OH with peak assignments.

^1^H NMR spectra of all polymer films were recorded, thus confirming chemical structure. Figure 7 and Figure 8 depict the ^1^H NMR spectra of 3238-3PDO and starting Priplast 3238 polyol and 3238-3POSS and starting POSS-OH, respectively, with peak assignments.

^1^H NMR spectra provided structural information regarding Priplast 3238 polyol, pure POSS-OH, and WPUD films. The main peaks of Priplast 3238 were in good agreement with those reported by Bueno-Ferrer’s work [12], showing a CH_2_C(O)O signal at 2.30 ppm and peaks from CH_2_OC(O) and CH_2_ in the β position of the ester group at 4.15 and 1.61, respectively (Figure 7).

The CH_2_ peaks from the methylene in the α, β, and γ position with respect to free hydroxyl groups (COO-C_γ_H_2_-C_β_H_2_-C_α_H_2_-OH) appeared at 3.69, 1.96, and 4.23 ppm, respectively. In the WPUD, the full conversion of hydroxyl groups to form polyurethane segments was confirmed by downfield shifting of the α and β peaks in the polymer spectra (Figure 7).

Commercially available IPDI consists of an isomer mixture of approximately 75:25 in favor of the cis-isomer, leading to different reaction mixtures [13] and resulting in complex ^1^H-NMR spectra. For a better interpretation of the ^1^H-NMR spectra of the synthesized polyurethane-ureas, they were compared with the ones of model compounds reported in our previous work [14] (IPDI:DMBA, IPDI:PDO, IPDI:EDA, IPDI:EtOH) that were obtained by reacting IPDI with the corresponding building blocks in a molar ratio of 2:1. This made it possible to more precisely assign some of the main signals of the polyurethane-ureas under study.

Urethane moieties gave the following weak signals:

- IPDI CH_2_ in α-position to -NHC(O) group (a’), 3.24 ppm and 2.88 ppm corresponding to the trans-isomer (25% abundance) and cis-isomer (75% abundance), respectively.
- IPDI CH in α-position to -NHC(O) group (g’), 3.73 ppm.

The incorporation of internal emulsifier (DMBA) into the polymer backbone could be assessed by the following signals: DMBA CH_2_ in α-position to OC(O) group (D1), 4.26 ppm; DMBA methylene group (D2) attached to methyl, 1.31 ppm; DMBA methyl group (D3), 0.86 ppm.

Triethylammonium salt could be assessed by the peaks at 3.02 (T1) and 1.37 (T2) ppm that correspond to CH_2_ and CH_3_, respectively, of the ethyl group.

Figure 8 depicts the ^1^H NMR spectra of sample 3238-3POSS and pure POSS-OH with peak assignments. Hydrogens 6, 6′ and 7, 7′ from the cyclohexyl ring were diastereotopic and therefore appeared at different chemical shifts. In pure POSS-OH, the signals from the methine hydrogens directly bonded to the hydroxyl groups appeared in the form of complex multiplets at 3.58 and 3.44 ppm. In all the WPUD containing POSS-OH as a building block, the full conversion of hydroxyl groups from POSS-OH to form polyurethane segments was confirmed by downfield shifting of the -CH-O- signals from the cyclohexyl ring in the polymer spectra (Figure 8).

**Figure 9:**
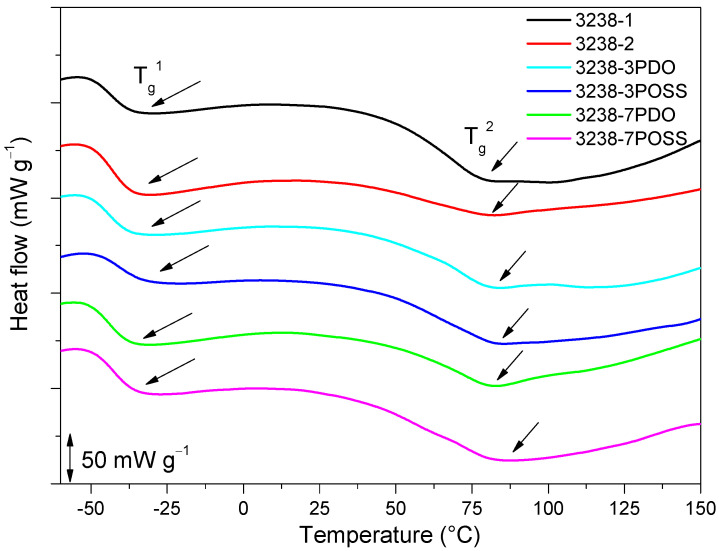
DSC thermogram of all of the synthesized WPUD. Glass-transition temperatures were calculated as the inflection point of the heating step scan DSC.

The thermal properties of the developed WPUD were analyzed by DSC and TGA. The non-isothermal DSC thermograms for all of the WPUD films as well as Priplast 3238 were registered. DSC curves of the synthesized WPUD performed at a heating rate of 10 °C min^−1^ are shown in Figure 9 and thermal transition temperatures for all of the WPUD are summarized in Table 4.

Two *T*_g_ values were detected for all of the synthesized WPUD, *T*_g_^1^ and *T*_g_^2^, which corresponded to soft segment and hard segment phases, respectively. The *T*_g_^1^ of the soft segment phase was close to the value observed for Priplast 3238 polyester polyol. This is an indication that there was a phase segregation between hard polyurethane-urea segments and polyester soft segments. All of the WPUD showed *T*_g_^2^ values between 70 and 90 °C, corresponding to the softening of hard segments. Melting temperatures and small enthalpies associated with the melting of crystallized hard segments were also detected in the range of 144–170 °C and data collected from these thermograms are shown in Table 4.

**Figure 10:**
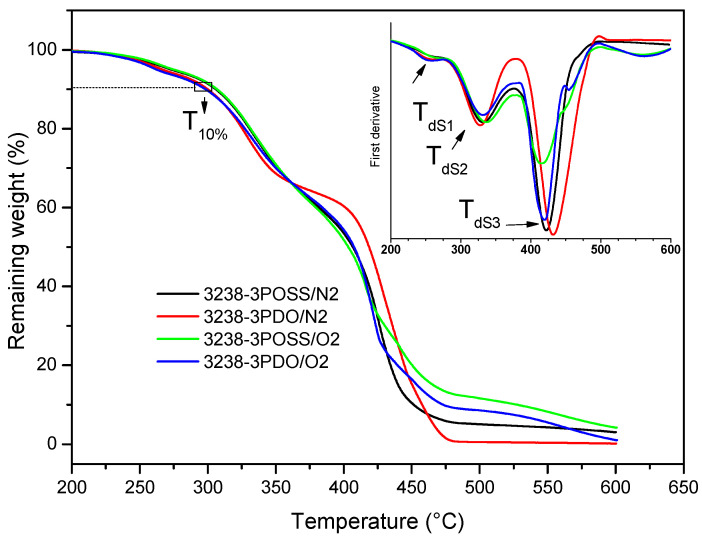
TGA thermograms of experiments 3238-3POSS and 3238-3PDO under a nitrogen atmosphere (/N2) and under an air atmosphere (/O2). DTGA curves are shown in inset graph.

The thermal stability of the synthesized WPUD films was studied via TGA under N_2_ and air atmospheres and the curves of experiments 3238-3POSS and 3238-3PDO are shown in Figure 10. The degradation temperatures under the N_2_ atmosphere corresponding to a weight loss of 10% and the temperatures of the maximum degradation rate for each degradation stage are collected in Table 4 for all of the WPUD. All of the (co)polymers had sufficient thermal stability to withstand, without degradation, the temperature conditions that are required during coating procedures.

As can be observed in Figure 10 and Table 4, under N_2_ conditions thermal degradation occurred in three stages, with similar temperatures of maximum degradation rate for all the synthesized WPUD. The first step occurred in the temperature range of 242–263 °C and could be attributed to the volatilization of triethylamine, which was in the form of carboxylate salt. Stage 2 took place in the temperature range of 307–332 °C and corresponded to the degradation of urethane and urea bonds [8,14]. Finally, Stage 3, between 416 and 432 °C, was related to the degradation of segments of Priplast 3238 polyol as far as the TGA curve of this pure polyol showed a single degradation step under an N_2_ atmosphere at a maximum degradation rate of 414 °C. Thermal degradation profiles under N_2_ and air atmospheres were quite similar except for the fact that a fourth degradation step around 550 °C could be detected under oxidative conditions due to the complete decomposition to CO_2_. It is also worth noting that polymers containing POSS-OH in their structure left a residual weight that corresponded with silicon dioxide (Figure 10).

**Figure 11:**
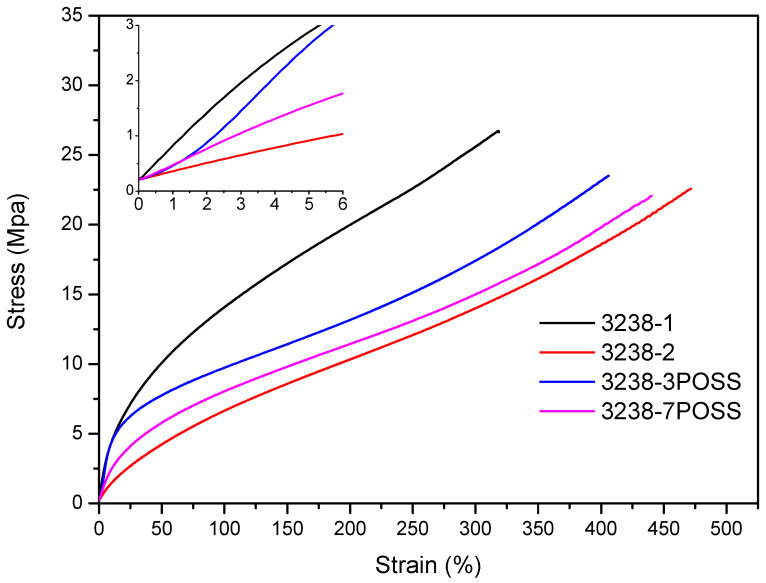
Stress–strain curves of WPUD films from experimental design that included POSS-OH in the ternary mixture.

**Figure 12:**
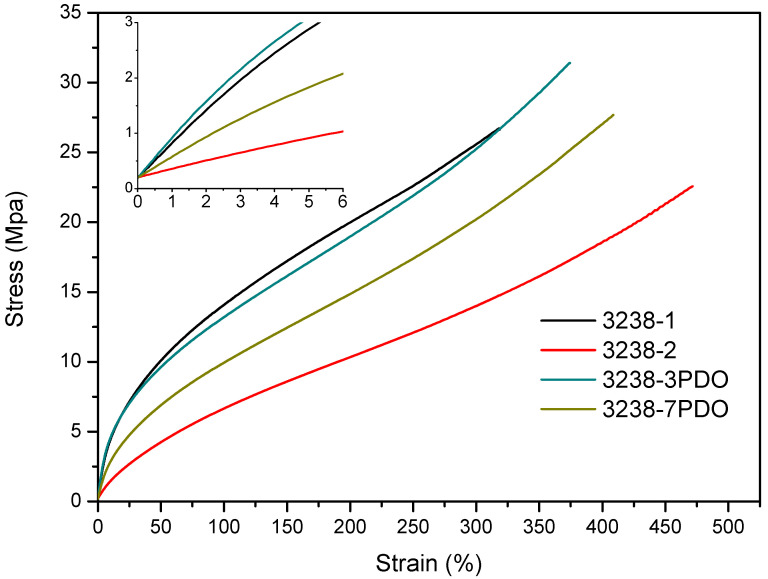
Stress–strain curves of WPUD films from experimental design that included PDO in the ternary mixture.

Stress–strain tensile experiments were carried out to evaluate the mechanical properties of the synthesized WPUD films. Figure 11 and Figure 12 display the stress–strain curves from the experimental designs that included POSS-OH and PDO in the tertiary mixture, respectively, and Table 5 displays the main mechanical properties of films.

**Figure 13:**
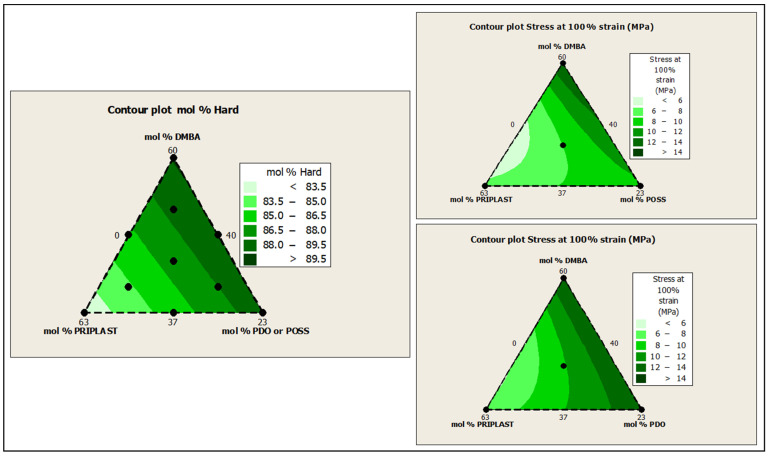
Contour plots obtained using the Minitab software from the ternary mixture experimental design. **Left**: contour plot of hard segment content in mol%. **Upper right**: contour plot of σ_100%_ (stress at 100% strain) for the ternary mixture design with POSS-OH. Bottom right: contour plot of σ_100%_ for the ternary mixture design with PDO.

**Figure 14:**
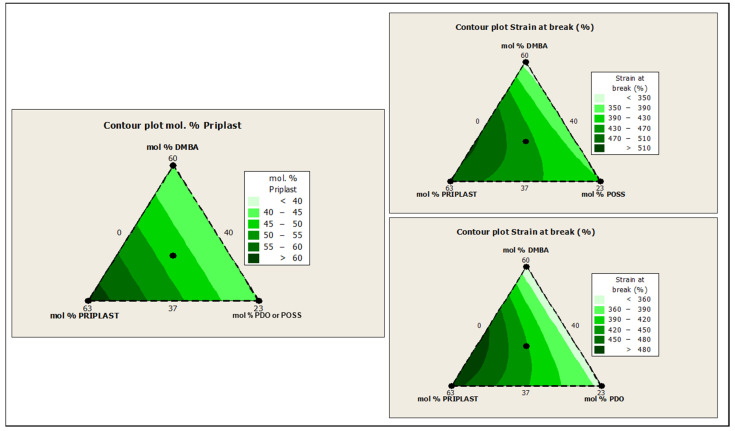
Contour plots obtained using the Minitab software from the ternary mixture experimental design. **Left**: contour plot of Priplast content (soft segment) in mol%. **Upper right**: contour plot of ε_b_ (strain at break) for the ternary mixture design with POSS-OH. Bottom right: contour plot of ε_b_ for the ternary mixture design with PDO.

The ratio of Priplast 3238, DMBA, and PDO or POSS-OH in the polyol ternary mixture determined the polyol composition in each synthesized polymer and, therefore, the hard segment content. There was a reasonably good match between the Young modulus and hard segment content. In general, higher hard segment content showed a higher Young modulus. However, experiment 3238-3PDO (38.8 wt.% HS) led to a slightly higher Young modulus than experiment 3238-1 (40.6 wt.% HS), which could be explained by the greater influence of PDO on the polymer rigidity caused by stronger hydrogen bonding interactions between urethane groups as compared to DMBA. Experiment 3238-3POSS (47.0 wt.% HS) led to a lower Young modulus than experiment 3238-1 (40.6 wt.% HS) due to the bulkiness of the heptaisobutyl POSS groups and the consequent reduction of hydrogen bonding interactions. The concordance between stress at 100% strain (σ_100%_) and hard segment content was even better, as can be seen in the surface plots in Figure 13. The strain at break was also very well correlated to the amount of Priplast 3238 (the polyol that provides flexibility); the higher the ratio of Priplast with respect to PDO or POSS-OH and DMBA, the higher the strain at break (see the contour plots in Figure 14).

**Figure 15:**
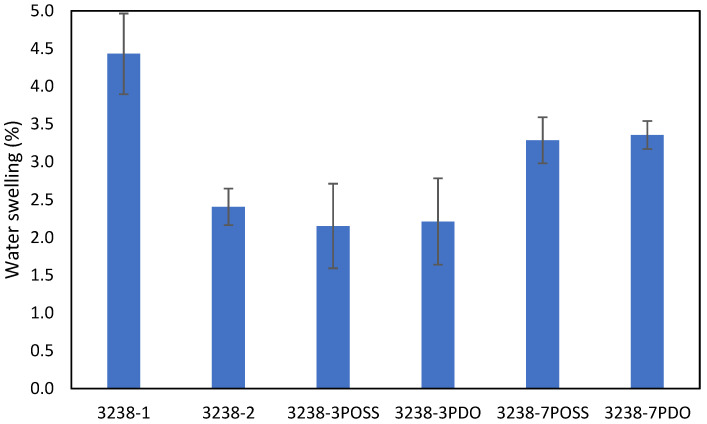
Swelling of WPUD films in water at 25 °C for 48 h.

**Figure 16:**
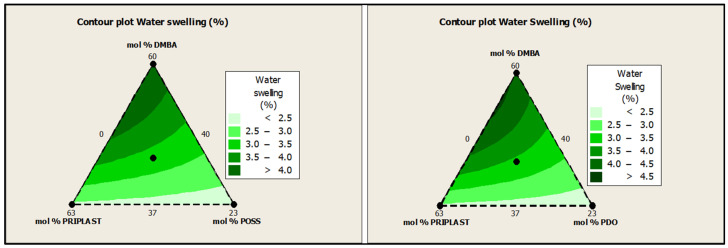
Contour plots of water swelling in wt.% of ternary mixture experimental design with POSS-OH (**left**) and PDO (**right**).

Water swelling of the films after 48 h at 25 °C was assessed on all of the WPUD films. Figure 15 shows the water swelling values. The swelling values of the films after 48 h were consistent with the increased content of hydrophilic chain extender (DMBA), in agreement with the behavior that was reported by Xu et al. for waterborne polyurethane emulsions using dimethylol propionic acid (DMPA) as an internal emulsifier [15]. Contour plots from the ternary mixture experimental designs clearly confirm the relationship between DMBA content and water swelling in Figure 16.

Dispersion stability, particle size, and particle size distribution of WPUD were also investigated. Figure S1 (Supplementary Material) shows the particle size distribution (PSD) curves of all developed aqueous polymeric dispersions analyzed by DLS. Particle size distribution curves are unimodal for all WPUD. Table 6 summarizes average particle size of all of the WPUD, PdI values, and Z-potential. Low particle size values ranging from 57 to 66 nm were observed for the dispersions with the lowest molar% of Priplast 3238, namely, 3238-1, 3238-3PDO, and 3238-3POSS. This is consistent with the fact that Priplast 3238 is a highly hydrophobic polyol of high Mw. Logically, the WPUD with the highest particle size was 3238-2 (203 nm) because it had the highest Priplast 3238 content. The dispersions with intermediate content in Priplast 3238 (3238-7PDO and 3238-7POSS) displayed intermediate particle size values. It is also worth mentioning that those WPUD with the same composition but having POSS-OH instead of PDO, for instance 3238-7POSS vs. 3238-7PDO, showed higher particle size, probably because POSS-OH provides more hydrophobicity than PDO and has a higher molecular weight. The polydispersity indexes (PdI) were below 0.2, which indicated that products had satisfactory stability and good dispersibility. The smaller the value of PdI, the better the homogeneity of the dispersion [16].

The stability of the dispersions was assessed by measuring the Z-potential of each WPUD. The Z-potential values are shown in Table 6. For all of the 3238 series, the Z-potential presented values ranging from −58 to −22 mV, indicating that the nanodroplets were negatively charged at the surface due to the presence of carboxylate groups. Most of the Z-potential absolute values were higher than 30 mV and were generally considered to represent stable emulsions [17,18]. However, 3238-1 and 3238-3PDO showed Z-potential absolute values below 30 mV. Despite this fact, the appearance of all of the WPUD emulsions stored at 4 °C and 40 °C for 6 months was good and there were no visual signs of instability such as phase separation, gelling, sedimentation, or creaming.

Finally, accelerated sedimentation tests of WPUD emulsions were also carried out. The analysis of the tested emulsions performed at 4 °C and 40 °C and relative centrifugal force of 2000× *g* is shown in Figures S2 and S3 (Supplementary Material). The first scanning profile obtained is indicated in red at the bottom, and the last in green at the top. Only a small clarification was observed at the meniscus area and small sedimentations were observed at the bottom with time, whereas the light transmission of the samples remained constant with time, indicating a good stability of the emulsions. The greater the change in light transmittance during the acceleration of the emulsion, the worse the stability [14].

### 3.2. Characterization of Textiles Coated with WPUD

In order to validate the applicability and properties of the polymeric dispersions, all of the synthesized WPUD were formulated in the form of printing pastes and subsequently face-coated on 100% polyester fabrics referenced as UPRON. Knife coating was the method chosen to carry out the coatings, as it is a widely employed method in the textile sector to produce accurate and reproducible coatings, being easily scalable to standard industrial textile machinery. In this way, this work has the purpose of performing the validation of the WPUD in application conditions that are very similar to those that are commonly used at an industrial level.

Smooth glass slides were also coated with all of the above-mentioned printing pastes, and WCA and OCA were measured to establish a comparison between the glass smooth coating substrate and the inherently rough textile substrate (UPRON).

Table 7 lists the values of WCA and OCA measured on glass slides and UPRON fabrics coated with WPUD printing pastes.

As could be observed, WCAs of coated UPRON fabrics were between 108 and 135° for the synthesized WPUD, which means low water wettability, but not superhydrophobicity (>150°). The incorporation of POSS-OH in the polymer increased significantly the WCA of the coated fabrics. It is important to point out that the WCAs of coated glass slides were much lower than the WCAs of WPUD coated fabrics. Thus, the inherent roughness of the textile substrate contributed to achieving high values of WCA [19]. The OCA values of the coated surfaces were low. However, it is worth noting that in the case of coated fabrics the oil droplets, while not maintaining their spherical shape, did not penetrate the coating either. This is an indication that the coatings exerted a remarkable barrier effect against olive oil.

The determination of resistance to water penetration measured by hydrostatic pressure tests (also known as water column), stiffness, air permeability, and water vapor resistance were performed on UPRON fabrics face-coated with the printing pastes (Table 8). Printing paste made from experiment 3238-2 showed the highest water column value of all the experiments, which is consistent with the mechanical properties of this sample, having the highest ε_b_ of all the samples. It is also worth noting that incorporation of POSS-OH in the polymer structure did not significantly alter water column properties with respect to polyurethane-ureas obtained without using POSS-OH as a co-polyol. Water column values higher than 30 cm were achieved by all of the coated fabrics, thus being in the same range as fossil-based coatings reported in our previous work [14].

Air permeability dramatically decreased and stiffness increased in all of the coated fabrics compared to untreated fabrics. This is logical when considering that we applied a polymer layer to one of the faces of the fabric. Reduced air permeability can be a positive feature when it comes to outdoor sportwear with wind-stopper functionality.

**Figure 17:**
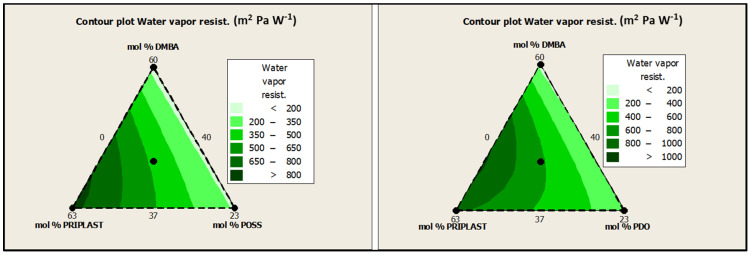
Contour plots of R_et_ for experimental design with POSS-OH (**left**) and experimental design with PDO (**right**).

The standard ISO 11092 uses the sweating guarded hotplate method (skin model method), to simulate the heat and moisture transfer process close to the human skin and to test the thermal resistance and water vapor resistance of textiles under steady-state conditions to evaluate the comfort of textiles. The lower the water vapor resistance (R_et_), the higher the breathability. In this work, R_et_ was assessed for all of the coated fabrics on the coated face (“face”) and on the uncoated face (“back”) with no significant differences between “face” and “back”. However, it should be noted that the greater the soft segment content, the greater the water vapor resistance, as can be clearly seen in contour plots from Figure 17. Although the R_et_ values obtained for all of the coatings were quite high (>150 m^2^ Pa W^−1^) and denoted low perspiration breathability and therefore low comfort, we can conclude that coating breathability decreased for those polymers with higher amounts of Priplast 3238. On the other hand, for a given mol.% of Priplast 3238 and DMBA, R_et_ decreased when we replaced PDO for POSS-OH (Ret 3238-3PDO > Ret 3238-3POSS, Ret 3238-7PDO > Ret 3238-7POSS). This observation made us think that the incorporation of bulky heptaisobutyl POSS as a pendant side group in the polymer chain prevented close packing of adjacent chains, thus favoring water molecule diffusion.

**Figure 18:**
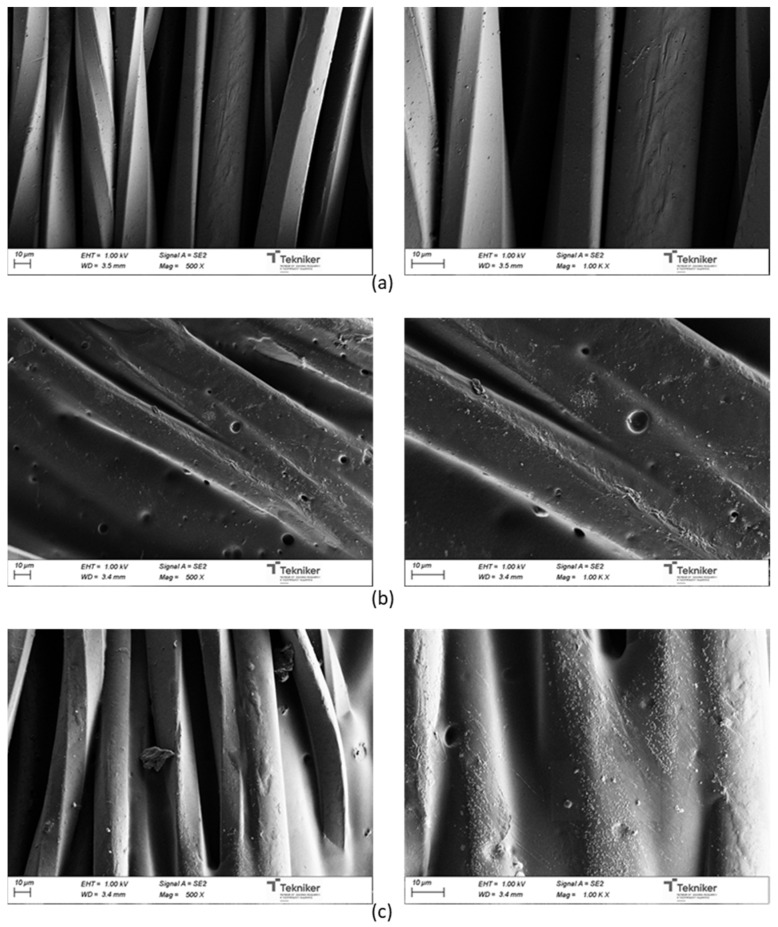
SEM micrographs for: (**a**) untreated UPRON and fabrics coated with (**b**) 3238-3PDO and (**c**) 3238-3POSS.

Figure 18 shows the SEM micrographs of uncoated and coated UPRON fabrics with the printing pastes made from experiments 3238-3PDO and 3238-3POSS. The presence of the coating in the fiber’s surface could be seen for coated samples. The coatings were not distributed in a completely homogeneous way. The roughness of the surfaces seemed to increase with the presence of the POSS-OH.

**Figure 19:**
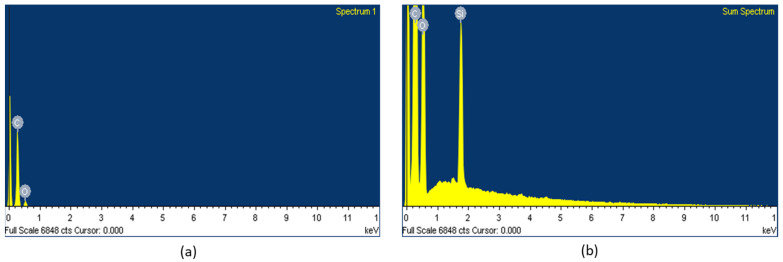
EDS spectra of fabrics coated with (**a**) 3238-3PDO and (**b**) 3238-3POSS.

The presence of the element Si in the surface could be clearly observed in the fabrics coated with WPUD containing heptaisobutyl POSS functional groups, as can be observed in the EDS spectra (Figure 19).

Finally, it is important to underline that all WPUD led to stable coating pastes with good runnability properties with the Werner Mathis coating machine. Therefore, it is to be expected that the WPUD would lead to good results in later stages of industrial scaling.

## 4. Conclusions

In this work, waterborne partially biobased polyurethane-urea dispersions have been developed, with a biobased content ranging from 53 to 72 wt.%. All the obtained WPUD were fluorine-free, low VOC, and showed proper mechanical and thermal properties that make them perfectly viable to be used as a coating for textile industry, providing coated fabrics with hydrophobic properties and a water column. Water contact angles close to 140° have been achieved by incorporating trans-cyclohexanediol isobutyl POSS into the polymer chain, a hybrid nanomaterial that has provided an important improvement in the hydrophobicity of the coatings without altering the mechanical, thermal or applicative properties of polyurethane-ureas. The characterization by FTIR and NMR showed that trans-cyclohexanediol isobutyl POSS has been covalently fixed in the polymer chain and therefore none of the known drawbacks are expected when working with discrete nanomaterials (bioavailability, toxicity, leachate, and liberation in the environment). Water column values higher than 30 cm have been achieved by all of the coated fabrics, thus being in the same range as fossil-based coatings reported in our previous work.

## Figures and Tables

**Table 1:** The simplex design table with experiments carried out in this work.

Experiment	Matrix				Molar%				Hard Segment (wt.%)	Biobased Content (wt.%)
	DMBA	Priplast 3238	POSS-OH	PDO	DMBA	Priplast 3238	POSS-OH	PDO		
3238-1	1	0	0	0	60.00	40.00	0.00	0.00	40.6	59.4
3238-2	0	1	0	0	37.00	63.00	0.00	0.00	28.0	72.0
3238-3POSS	0	0	1	0	37.00	40.00	23.00	0.00	47.0	53.0
3238-3PDO	0	0	0	1	37.00	40.00	0.00	23.00	38.8	62.6
3238-7POSS	1/3	1/3	1/3	0	44.67	47.67	7.67	0.00	37.9	62.1
3238-7PDO	1/3	1/3	0	1/3	44.67	47.67	0.00	7.67	35.0	65.4

**Table 2:** Printing paste formulation employed to perform the coating of the fabrics with WPUD.

Composition	Printing Paste Formulation (g)
WPUD	85
Water	15
Defoamer PR	1
Complex DG	2
DMEA	1
Thickener L-120	Added drop-by-drop under high shear until viscosity of 18,000 ± 50 cPs is reached (Brookfield RV 6/30)
Dry polyurethane-urea content	30 ± 1%

**Table 3:** Standards employed to evaluate the properties of the coated textiles.

Property	Standard
Determination of resistance to water penetration-Hydrostatic pressure test (water column)	EN 20811:1992
Fabric stiffness	ČSN 80 0858
Determination of the permeability of fabrics to air	ISO 9237:1995
Water vapor resistance under steady-state conditions (sweating guarded hotplate test)	ISO 11092:2014

**Table 4:** Thermal properties of WPUD films evaluated by DSC, TGA, and DMTA.

Reference	DSC ^a^				TGA ^b^			
	*T*_g_^1^ (°C)	*T*_g_^2^ (°C)	*T_m_* (°C)	Δ*H*_m_ (J/g)	*T*_10%_ (°C)	*T*_dS1_ (°C)	*T*_dS2_ (°C)	*T*_dS3_ (°C)
3238-1	−43	70	153	4.4	284	242	309	416
3238-2	−43	88	148	1.0	308	247	307	418
3238-3POSS	−40	77	149	3.1	306	255	333	423
3238-3PDO	−44	73	170	3.9	298	252	328	432
3238-7POSS	−43	74	144	2.3	308	263	332	430
3238-7PDO	−44	90	155	2.5	298	243	307	416
Priplast 3238	−59	-	-	-	403	-	-	414

^a^*T*_g_, *T*_m_, and Δ*H*_m_ values determined by DSC. ^b^ TGA characterization of the WPUD films under N_2_ atmosphere: *T*_10%_ is the temperature at which a 10 wt.% loss was observed in the TGA traces recorded at 10 °C min^−1^; *T*_dS1_, *T*_dS2_, and *T*_dS3_ are the temperatures of maximum degradation rate for first, second. and third degradation stages, respectively.

**Table 5:** Mechanical characterization of WPUD films.

Reference	E ^a^ (Mpa)	σ_100%_^a^ (Mpa)	σ_b_^a^ (Mpa)	ε_b_^a^ (%)
3238-1	59.0 ± 1.4	14.1 ± 0.2	27.5 ± 1.2	335.0 ± 13.0
3238-2	14.8 ± 0.3	6.6 ± 0.1	23.6 ± 1.8	490.0 ± 22.7
3238-3POSS	39.7 ± 4.9	9.6 ± 0.2	22.7 ± 1.1	391.8 ± 9.6
3238-3PDO	64.2 ± 1.3	13.3 ± 0.3	29.4 ± 1.6	352.7 ± 23.6
3238-7POSS	29.1 ± 0.4	8.0 ± 0.1	22.1 ± 0.5	443.0 ± 9.5
3238-7PDO	35.2 ± 1.6	9.8 ± 0.4	27.9 ± 1.7	422.3 ± 14.5

^a^ E: Young modulus, σ_100%_: stress at 100% strain, σ_b_: stress at break, ε_b_: strain at break.

**Table 6:** Summary of the average particle size, PdI, and Z-potential values for synthesized WPUD dispersions.

Reference	Average Particle Size (nm)	PdI	Z Potential (mV)	Storage Stability ^a^	
				4 °C	40 °C
3238-1	63 ± 21	0.15 ± 0.01	−23 ± 11	Good	Good
3238-2	203 ± 73	0.16 ± 0.01	−34 ± 7	Good	Good
3238-3POSS	66 ± 22	0.08 ± 0.01	−51 ± 19	Good	Good
3238-3PDO	57 ± 20	0.10 ± 0.01	−22 ± 10	Good	Good
3238-7POSS	158 ± 80	0.14 ± 0.01	−58 ± 12	Good	Good
3238-7PDO	99 ± 43	0.15 ± 0.01	−56 ± 16	Good	Good

^a^ Good storage stability means that sealed glass vials stored at the indicated temperature for 6 months did not show sedimentation, creaming of visible alteration of the original dispersion appearance.

**Table 7:** WCA and OCA of glass and UPRON substrates coated with WPUD.

Coating Reference	Substrate			
	Glass Slides		UPRON ^a^	
	WCA (°)	OCA (°)	WCA (°)	OCA (°)
Uncoated	Spreads	Spreads	Wets	Wets
3238-1	85 ± 2	22 ± 2	117 ± 1	37 ± 1
3238-2	90 ± 1	25 ± 1	112 ± 1	37 ± 1
3238-3POSS	93 ± 1	32 ± 2	135 ± 1	33 ± 1
3238-3PDO	84 ± 1	30 ± 1	108 ± 1	35 ± 2
3238-7POSS	97 ± 1	29 ± 1	132 ± 1	33 ± 1
3238-7PDO	84 ± 1	26 ± 1	117 ± 1	32 ± 2

^a^ The dry add-on of the coated UPRON fabrics was 26 ± 3 g m^−2^ for all the coated samples.

**Table 8:** Stiffness, water column, and air permeability of UPRON fabrics uncoated and coated by knife coating procedure.

Coating Reference	Dry Add-On (g/m^2^)	Stiffness [mN]		Water Column (cm)	Air Permeability (mm/s)	Water Vapor Resistance (R_et_ [m^2^ Pa W^−1^])	
		Warp	Weft			Face	Back
Uncoated	-	17.4	9.7	<15 soaked	85.3	3.6	3.6
3238-1	24.9	133.5	38.0	34.1	1.3	150.0	160.0
3238-2	28.9	122.0	36.3	36.2	0.8	841.0	760.0
3238-3POSS	24.0	110.0	37.5	31.0	1.3	184.0	190.0
3238-3PDO	25.5	132.5	44.0	31.3	1.2	292.0	274.0
3238-7POSS	27.1	110.0	44.1	31.7	0.5	469.0	448.0
3238-7PDO	24.0	124.7	41.4	35.3	0.7	631.0	667.0

## Data Availability

The raw/processed data required to reproduce these findings cannot be shared at this time as the data also forms part of an ongoing study.

## Supplementary Materials

The following are available online at https://www.mdpi.com/article/10.3390/polym13203526/s1. Figure S1: Particle size distribution of WPUD measured by DLS, (a) 3238-1, (b) 3238-2, (c) 3238-3POSS, (d) 3238-3PDO, (e) 3238-7POSS, (f) 3238-7PDO. Figure S2: LUM transmission profiles of WPUD measured at 470 nm, 4 °C, relative acceleration force (RCA) 2000× *g* (3900 R.P.M.). (a) 3238-1, (b) 3238-2, (c) 3238-3POSS, (d) 3238-3PDO, (e) 3238-7POSS, (f) 3238-7PDO. Figure S3: LUM transmission profiles of WPUD measured at 470 nm, 40 °C, relative acceleration force (RCA) 2000× *g* (3900 R.P.M.). (a) 3238-1, (b) 3238-2, (c) 3238-3POSS, (d) 3238-3PDO, (e) 3238-7POSS, (f) 3238-7PDO.

## References

[1] Luz A.L., Anderson J.K., Goodrum P., Durda J. (2019). Perfluorohexanoic acid toxicity, part I: Development of a chronic human health toxicity value for use in risk assessment. Regul. Toxicol. Pharmacol..

[2] Knight E.R., Bräunig J., Janik L.J., Navarro D.A., Kookana R.S., Mueller J.F., McLaughlin M.J. (2021). An investigation into the long term binding and uptake of PFOS, PFOA and PFHxS in soil–plant systems. J. Hazard. Mater..

[3] Schellenberger S., Hill P.J., Levenstam O., Gillgard P., Cousins I.T., Taylor M., Blackburn R.S. (2019). Highly fluorinated chemicals in functional textiles can be replaced by re-evaluating liquid repellency and end-user requirements. J. Clean. Prod..

[4] Chinthapalli R., Skoczinski P., Carus M., Baltus W., De Guzman D., Käb H., Raschka A., Ravenstijn J. (2019). Biobased Building Blocks and Polymers-Global Capacities, Production and Trends, 2018–2023. Ind. Biotechnol..

[5] Coates G.W., Hillmyer M.A. (2009). A Virtual Issue of Macromolecules: “Polymers from Renewable Resources”. Macromolecules.

[6] Santamaria-Echart A., Fernandes I., Barreiro F., Corcuera M.A., Eceiza A. (2021). Advances in Waterborne Polyurethane and Polyurethane-Urea Dispersions and Their Eco-friendly Derivatives: A Review. Polymers.

[7] Cavallo D., Gardella L., Soda O., Sparnacci K., Monticelli O. (2016). Fully bio-renewable multiblocks copolymers of poly(lactide) and commercial fatty acid-based polyesters polyols: Synthesis and characterization. Eur. Polym. J..

[8] Poussard L., Lazko J., Mariage J., Raquez J.M., Dubois P. (2016). Biobased waterborne polyurethanes for coating applications: How fully biobased polyols may improve the coating properties. Prog. Org. Coat..

[9] Lai Y.S., Tsai C.W., Yang H.W., Wang G.P., Wu K.H. (2009). Structural and electrochemical properties of polyurethanes/polyhedral oligomeric silsesquioxanes (PU/POSS) hybrid coatings on aluminum alloys. Mater. Chem. Phys..

[10] Madbouly S.A., Otaigbe J.U., Nanda A.K., Wicks D.A. (2007). Rheological Behavior of POSS/Polyurethane-Urea Nanocomposite Films Prepared by Homogeneous Solution Polymerization in Aqueous Dispersions. Macromolecules.

[11] Poussard L., Mecheri A., Mariage J., Barakat I., Bonnaud L., Raquez J.M., Dubois P. (2014). Synthesis of oligo(butylene succinate)-based polyurethanes: Influence of the chemical structure on thermal and mechanical properties. J. Renew. Mater..

[12] Bueno-Ferrer C., Hablot E., Perrin-Sarazin F., Garrigós M.C., Jiménez A., Averous L. (2012). Structure and Morphology of New Bio-Based Thermoplastic Polyurethanes Obtained From Dimeric Fatty Acids. Macromol. Mater. Eng..

[13] Lomölder R., Plogmann F., Speier P. (1997). Selectivity of isophorone diisocyanate in the urethane reaction influence of temperature, catalysis, and reaction partners. J. Coat. Technol..

[14] Lacruz A., Salvador M., Blanco M., Vidal K., Goitandia A.M., Martinková L., Kyselka M., de Ilarduya A.M. (2021). Biobased Waterborne Polyurethane-Urea/SWCNT Nanocomposites for Hydrophobic and Electrically Conductive Textile Coatings. Polymers.

[15] Xu J., Li T., Zhao W., Li P., Wu Y. (2016). Synthesis and characterization of waterborne polyurethane emulsions based on poly(butylene itaconate) ester. Des. Monomers Polym..

[16] Sun Y., Zhao X., Liu R., Chen G., Zhou X. (2018). Synthesis and characterization of fluorinated polyacrylate as water and oil repellent and soil release finishing agent for polyester fabric. Prog. Org. Coat..

[17] Li D., Müller M.B., Gilje S., Kaner R.B., Wallace G.G. (2008). Processable aqueous dispersions of graphene nanosheets. Nat. Nanotechnol..

[18] Sheng L., Zhang X., Ge Z., Liang Z., Liu X., Chai C., Luo Y. (2018). Preparation and properties of waterborne polyurethane modified by stearyl acrylate for water repellents. J. Coat. Technol. Res..

[19] Lacruz A., Salvador M., Blanco M., Vidal K., Martínez de Ilarduya A. (2021). Development of fluorine-free waterborne textile finishing agents for anti-stain and solvent-water separation based on low surface energy (co)polymers. Prog. Org. Coat..

