# Supplementary material for: Biobased Waterborne Polyurethane-Ureas Modified with POSS-OH for Fluorine-Free Hydrophobic Textile Coatings
Source: Polymers (Basel). 2021 Oct 13;13(20):3526. doi: 10.3390/polym13203526 (PMC8537187; doi:10.3390/polym13203526)
Supplement: Supplementary file 1 [file polymers-13-03526-s001.zip › polymers-1411368-SM.pdf]

polymers

MDPI

Supplementary Material: Biobased Waterborne Polyurethane-ureas Modified with POSS-OH for Fluorine-free Hydrophobic Textile Coatings

Amado Lacruz, Mireia Salvador, Miren Blanco, Karmele Vidal, Amaia M. Goitandia, Lenka Martinková, Martin Kyselka, and Antxon Martínez de Ilarduya

1. Particle size distribution of WPUD

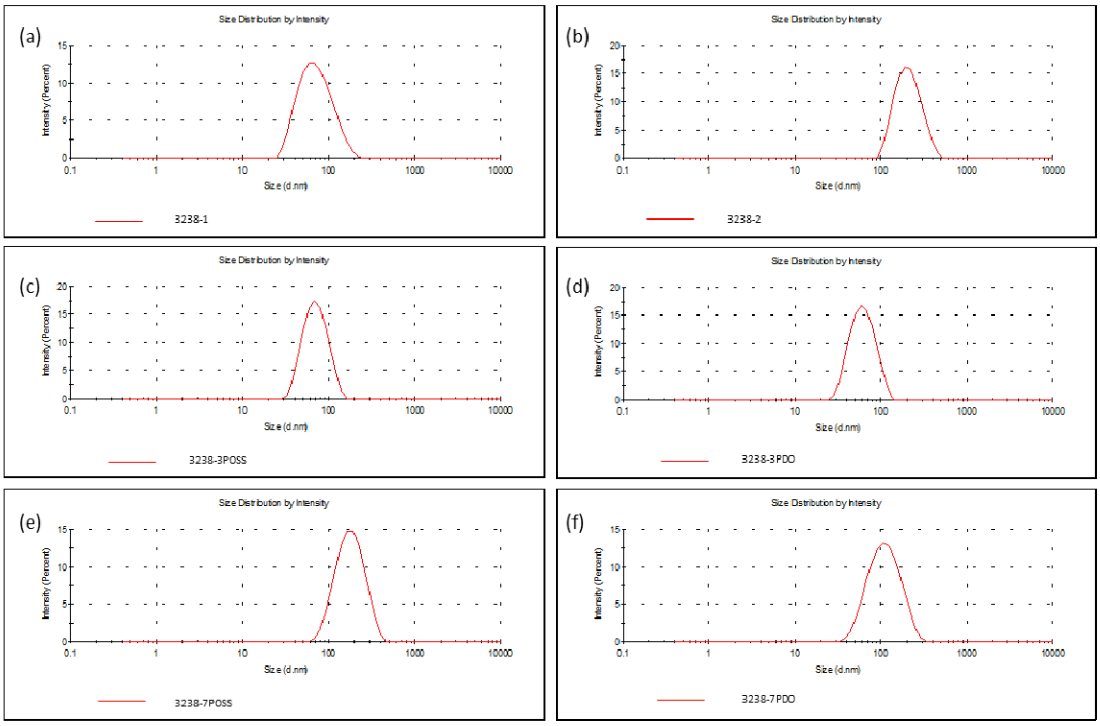

Figure S1. Particle size distribution of WPUD measured by DLS, (**a**) 3238-1, (**b**) 3238-2, (**c**) 3238-3POSS, (**d**) 3238-3PDO, (**e**) 3238-7POSS, (**f**) 3238-7PDO.

2. LUM measurements of WPUD

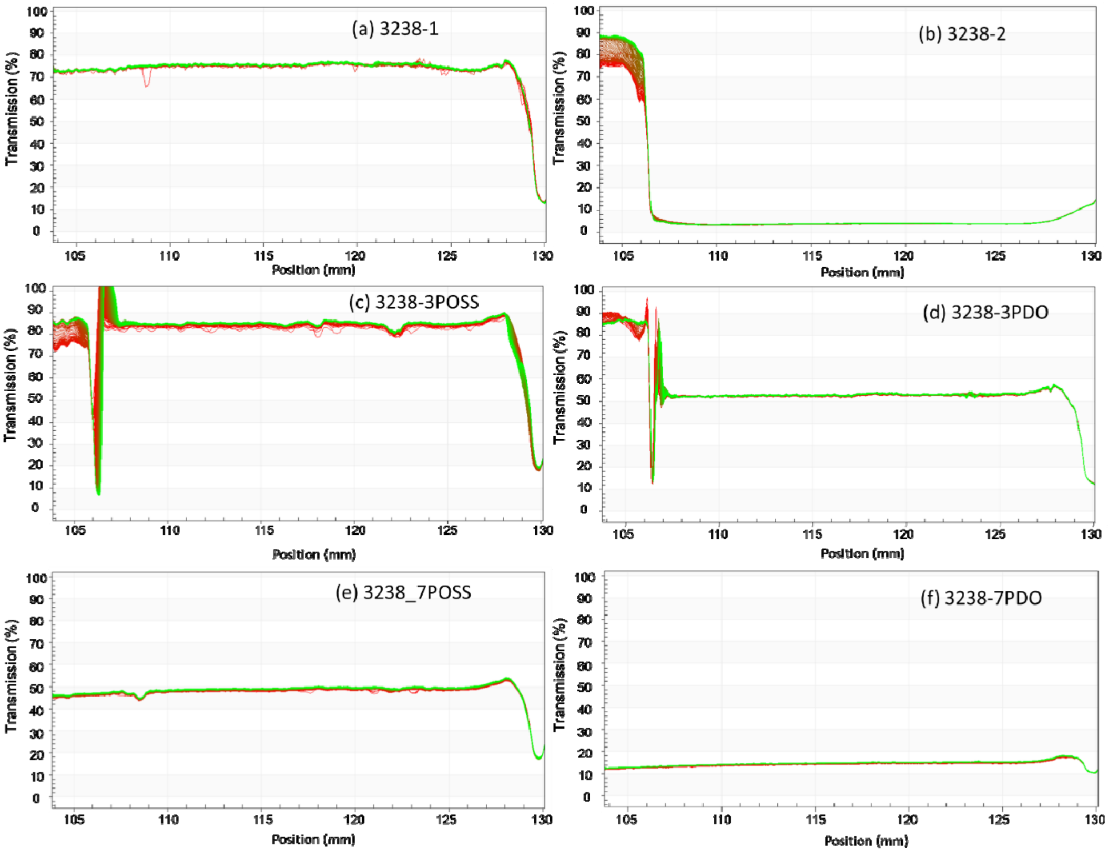

Figure S2. LUM transmission profiles of WPUD measured at 470 nm, 4 °C, Relative acceleration force (RCA) 2000 x g (3900 R.P.M.). (**a**) 3238-1, (**b**) 3238-2, (**c**) 3238-3POSS, (**d**) 3238-3PDO, (**e**) 3238-7POSS, (**f**) 3238-7PDO.

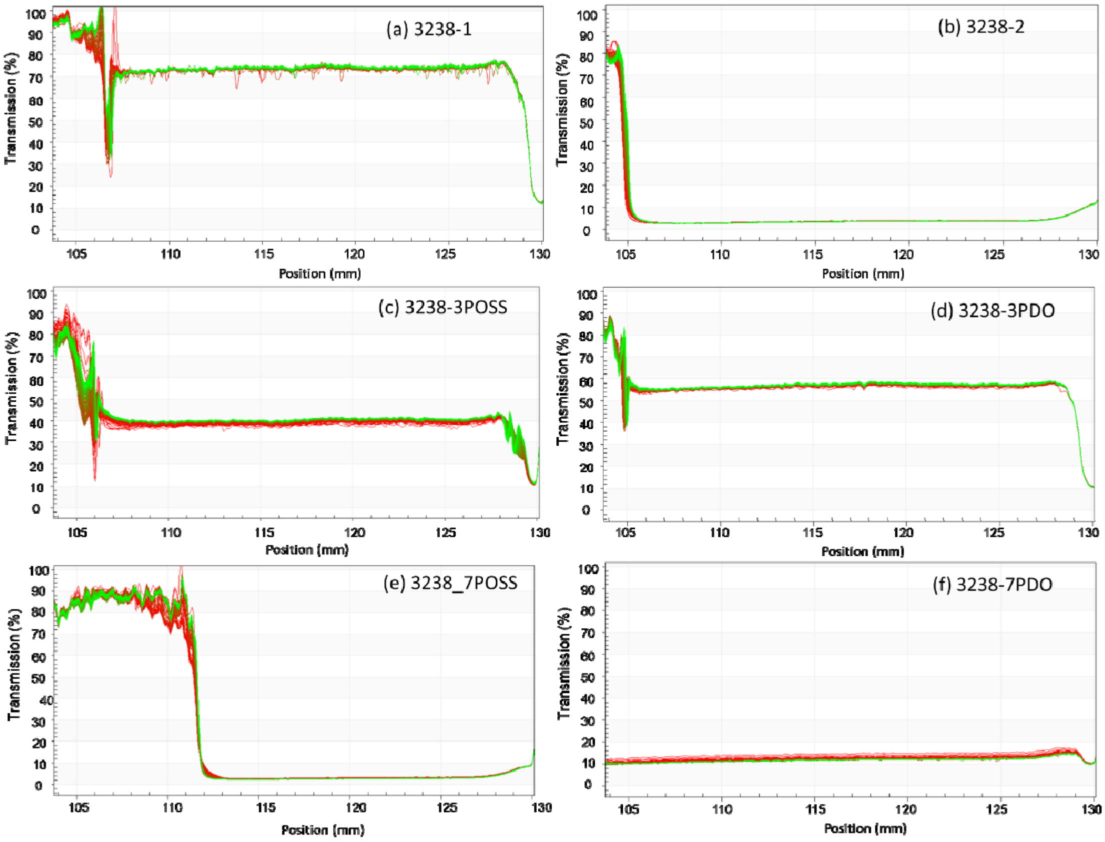

Figure S3. LUM transmission profiles of WPUD measured at 470 nm, 40 °C, Relative acceleration force (RCA) 2000 x g (3900 R.P.M.). (**a**) 3238-1, (**b**) 3238-2, (**c**) 3238-3POSS, (**d**) 3238-3PDO, (**e**) 3238-7POSS, (**f**) 3238-7PDO.
